# The role of mating effort and co-residence history in step-grandparental investment

**DOI:** 10.1017/ehs.2024.17

**Published:** 2024-05-16

**Authors:** Jenni E. Pettay, David A. Coall, Mirkka Danielsbacka, Antti O. Tanskanen

**Affiliations:** 1 INVEST Research Flagship Centre, University of Turku, Turku, Finland; 2 Department of Social Research, University of Turku, Turku, Finland; 3 School of Medical and Health Sciences, Edith Cowan University, Joondalup, Australia; 4 Population Research Institute, Väestöliitto, Helsinki, Finland

**Keywords:** intergenerational relations, step-family, grandchildren, step-parent, step-children

## Abstract

The prevalence of divorce in both parental and grandparental generations has led to a rise in the number of children who now have families that include both biological and step-grandparents. Despite the thorough examination of biological grandparents’ contributions in the recent literature, there remains a scarcity of studies focusing on the investment of step-grandparents. Using population-based data from a sample of 2494 parents in Germany, we assessed grandparental investment through financial support and assistance with childcare of grandparents (*N* = 4238) and step-grandparents (*N* = 486). The study revealed that step-grandparents provided lower levels of investment in their grandchildren compared with biological grandparents. Furthermore, the study identified that a longer duration of co-residence between step-grandparents and parents earlier in life did not correspond to an increase or decrease in step-grandparental investment. However, investment by separated biological grandparents increased with the increasing length of co-residence with parents. In line with the scarce literature on step-grandparental investment, these findings indicate that mating effort may be the most important motivation for step-grandparental investment.

## Graphical abstract

**Figure:**
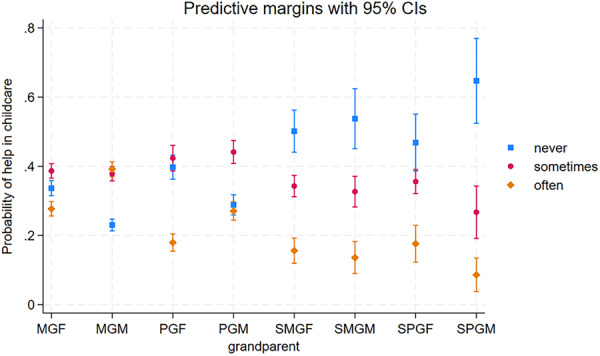

**Social media summary:** The step-gap in grandparental investment was not mitigated by childhood co-residence with the middle-generation parents.

## Introduction

Everyone possesses exactly four biological grandparents, regardless of how much time they are together and activities they share. The rise in divorce and re-marriage rates in both parental and grandparental generations has meant that children increasingly also have step-grandparents in their lives. Consequently, children often have additional grandparents, beyond the more commonly studied biological grandparents. Indeed, owing to these demographic changes, in some cases grandchildren may have more contact with their step-grandparents than biological grandparents (Daly & Perry, 2021). There are multiple pathways to becoming a step-grandparent, and three main ways can be identified (Ganong & Coleman, 2004; Pashos et al., 2016; Steinbach & Silverstein, 2020). A step-grandparent can be characterised as (a) the step-parent of a parent, (b) the parent of a step-parent or (c) the step-parent of a step-parent. For the purposes of the current study, our focus will be on the first-mentioned type of step-grandparent, which refers to being the step-parent of a parent. This means that in our study, step-grandparenting is viewed as a continuation of step-parenting after the birth of a grandchild.

In line with inclusive fitness theory (Hamilton, 1964), *step-parents* are consistently found to invest less in children compared with biological parents (e.g. Anderson, 2011; Arat et al., 2022; Pettay et al., 2023). This has been referred to as the ‘step-gap’ (e.g. Becker et al., 2013; Delongis & Preece, 2002) and ‘Cinderella effect’ (e.g. Daly & Perry, 2021) in prior literature. Step-parenting is not a novelty among humans, and the relationship between step-parents and step-children can extend beyond childhood (Kalmijn, 2013; Becker et al., 2013; Pettay et al., 2023). The origin of step-parental investment, i.e. investment in non-biological offspring, is thought to be related more to mating behaviours than parenting behaviour, within an evolutionary framework. Stepparents demonstrate their commitment to their current partner by investing in their partner's offspring from a previous relationship (Rohwer et al., 1999). In the present study, we explore grandparental investment, which can be viewed as an extension of parental investment (Coall & Hertwig, 2010).

Grandparents are found to invest heavily in their grandchildren across societies (Coall & Hertwig, 2010; Sear & Mace, 2008). With that said, however, all grandparents do not invest equally in their grand offspring. The most robust pattern of investment by biological grandparents appears to be that maternal grandmothers invest the most, followed by maternal grandfathers, paternal grandmothers and finally paternal grandfathers (e.g. Euler & Weitzel, 1996; Euler, 2011; Danielsbacka et al., 2011; Laham et al., 2005; Pollet et al., 2006, 2007). To date, a handful of studies have investigated differences in the investment by both biological and step-grandparents. A set of studies using small or non-representative samples have detected a ‘grand step-gap’, i.e. step-grandparents invest less in grandchildren than do biological grandparents (e.g. Block, 2002; Christensen & Smith, 2002; Gray & Brogdon, 2017; Henry et al., 1992; Pashos et al., 2016; Sanders & Trygstad, 1989; Soliz, 2007). These findings are supported by the few studies that have used large and representative data sources; therefore, it appears that step-grandparents consistently invest less than biological grandparents in their grandchildren (Coall et al., 2014; Daly & Perry, 2021; Steinbach & Silverstein, 2020; Tanskanen et al., 2014, 2020).

The mating effort hypothesis predicts that step-parents invest in their step-children as a means of investing in their current relationship with the children's biological parent. Owing to their advanced age, step-grandparents are typically no longer capable of reproduction, meaning that investment in step-grandchildren seldom leads to direct reproductive benefits. In the case of humans, however, mating effort can also extend beyond reproductive endeavours, as individuals often seek supportive, engaging relationships without intentions of reproduction (Anderson et al., 1999).

Another factor associated with care between family members is the legth of co-residence. A child co-residing for longer with another family member is generally associated with increased investment by kin in each other and may also explain changes in grandparental investment patterns. For instance, an increased co-residence duration in childhood is associated with increased investment by step-fathers and fathers who had separated from the mother (e.g. Kalmijn, 2013; Pettay et al., 2023) and half-siblings (e.g. Tanskanen & Danielsbacka, 2019; Tanskanen et al., 2021; Lieberman et al., 2007; Steinbach & Hank, 2018). To the best of our knowledge, however, no studies have tested whether childhood co-residence duration between step-grandparents and the middle-generation's parents is associated with the investment step-grandparents channel towards grandchildren. Here, we propose that childhood co-residence might hold even greater significance for step-grandparents compared with biological grandparents because biological grandparents have an underlying fitness-based reason for investing in their children after divorce. Step-grandparents, however, who do not have any biological relationship with the grandchild, may receive their signal for increasing investment from their close association during childhood. Living together in the same household allows children and step-parents to develop a ‘kin-like’ connection, potentially leading them to see each other as emotional kin (Rotkirch, 2018). As a result, childhood co-residence can enhance the psychological attachment between step-children and step-parents. It is an open question, however, whether this kin-like relationship pattern extends to the next generation, i.e. do step-grandparents also invest more in their grandchildren if they have co-resided in the same household with the grandchild's parent during their childhood?

## Hypotheses

In this study, we explore predictions based on mating effort and co-residence history. Subsequently, the following hypotheses are subjected to testing:

**Hypothesis 1 (H1):** Greater grandparental investment is made by biological grandparents compared with step-grandparents.

**Hypothesis 2 (H2):** An extended co-residence duration between step-grandparents and parents of the middle generation is associated with heightened step-grandparental investment in grandchildren.

**Hypothesis 3 (H3):** An extended co-residence duration between separated biological grandparents and the middle generation's parents is associated with increased grandparental investment in grandchildren.

## Data and methods

### Data

We used survey data from the Panel Analysis of Intimate Relationships and Family Dynamics (Pairfam), which offers information on intergenerational relations, childbearing and several socio-ecological factors in Germany (Brüderl et al., 2019a; Huinink et al., 2011). Pairfam provides longitudinal data on three birth cohorts born in 1971–1973, 1981–1983 and 1991–1993. We used the birth cohorts 1971–1973 and 1981–1983 of wave 2 data collected from 2010 to 2011, when the cohort members were aged approximately 27–29 and 37–39 years, respectively. This specific wave of data was used because questions concerning both childhood living arrangements and grandparental investment were recorded only in this wave.

### Measures

Grandparental investment, the dependent variable, was taken from the intergenerational relations section of the Pairfam questionnaire. Respondents (i.e. middle-generation) were asked questions about their relationships with their parents. We call respondents’ parents ‘grandparents’ to signify these different generations. Grandparents include both biological parents (even after separation) and step-parents when they existed. The questionnaire included two questions related to investment towards children of the parental generation. These questions were asked of the anchor respondent (parent) who had a child that was a biological child or lived with the respondent in the same household, and was under 15 years of age. *Financial support* is based on a question for each parent: ‘During the past 12 months, did you receive from (step)parent substantial gifts or financial support for your children?’ This response ranged from (0) never, (1) seldom, (2) sometimes and (3) often to (4) very often. *Help in childcare* is based on a question for each parent: ‘During the past 12 months, how often did you receive help from (step)parent in looking after or taking care of your children?’ This response ranged from (0) never, (1) seldom, (2) sometimes and (3) often to (4) very often. For analytical purposes and to ensure adequate sample sizes for each response category for each grandparent type, we combined 1 (seldom) and 2 (sometimes) into one category *sometimes*, and (3) often and (4) very often into one category *often*, resulting in three categories: never, sometimes and often for financial support and help in childcare.

The grandparental generation included respondents’ birth mother, birth father, step-father and step-mother. Step-grandparents were defined as partners of birth parents. If adult children had both step-fathers and step-mothers, the questions were limited to step-fathers only (see Brüderl et al., 2019b). They may have been the parent's partner already when respondents were children, or they might have become partners of the birth parent later in life. Because maternal and paternal grandparents are often found to invest differentially, grandparents were divided into eight grandparent type groups by parent type (mother, father, step-mother and step-father) and respondent's sex to distinguish maternal and paternal lineages. The eight grandparent type categories are: maternal grandfather (MGF), maternal grandmother (MGM), paternal grandfather (PGF), paternal grandmother (PGM), maternal step-grandfather (MSGF), maternal step-grandmother (MSGM), paternal step-grandfather (PSGF) and paternal step-grandmother (PSGM).

Childhood co-residence durations with birth parents were determined from questions about living arrangements before the age of 18 years. Shared physical custody is rare in Germany and after parental divorce children are much more likely to live with their mother than their father (Walper et al., 2021). For birth parents, co-residence duration was the number of years in which the respondent lived in the same household with their daughter or son until the age of 18. We assumed that the last step-parent the respondent lived with during childhood would be the corresponding step-parent at the time of survey; hence, childhood co-residence duration was calculated as 18 years minus the age when the respondent started living with the step-parent.

Other factors potentially influencing grandparental investment that were included in the models, when available, were cohort (two levels: 1971–1973 and 1981–1983), ethnicity (two levels: German native and other background based on the parents’ birth country), respondent's education based on ISCED-97 classification (ranging from primary and lower secondary to tertiary education (currently enrolled were grouped with primary and lower secondary)), cohabiting status (cohabiting or not cohabiting with someone), number of biological children cropped at three owing to relatively small numbers of more than three children (one, two and three or more children) and age of the youngest child in years (continuous). Variables related to grandparent are travelling distance to the residence of the grandparent (0 = living in the same household, 1 = less than 10 minutes, 2 = 10 minutes to less than 30 minutes, 3 = 30 minutes to less than 1 hour, 4 = 3 hours or more), and grandparent's cohabitation status (yes/no). Unfortunately, data on (step-)grandparents’ health, income and number of other grandchildren was not available. We did not include grandstep-parents’ age, because the grandparents’ age is correlated with the cohort and preliminary analysis suggests that grandparental age is not related to either financial support or help in childcare. Further, step-grandparents were more likely to have missing age: 53% for step-grandmothers in contrast to 6% for grandmothers and 36% for step-grandfathers and 7% for grandfathers, probably because respondents were not aware of their step-parents ages. For other explanatory variables the frequency of missing values was reasonable (ranging from 0 to 3.9%, missing 1% on average) and therefore list-wise deletion was used. Pairfam includes a design weight to correct for disproportionate sampling across cohorts (Brüderl et al., 2019b). Including this weight did not alter our results and we present these results only in the Supporting Information (supplementary Tables S3–S8). Descriptive statistics of the respondents’ data (*N =* 4,724) are shown in Table 1. For the purpose of analyses, we reshaped the data from the perspective of the (step)grandparent, with each (step)grandparent being an observation, producing up to three observations per respondent. Descriptive statistics for grandparents are shown in Table 2.

**Table 1.** Descriptive statistics for the respondents (parents)

Variable	Frequency/mean	SD
*Sex*		
Male	865	
Female	1629	
*Cohort*		
1981–1983	691	
1971–1973	1803	
*Ethnicity*		
German native	1838	
Other countries	656	
*Education*		
Primary and lower secondary	264	
Upper secondary	1220	
Post secondary	287	
Tertiary	723	
*Cohabitation status*		
(Not cohabiting 0)	313	
(Cohabiting 1)	2181	
*Number of biological children*		
1	955	
2	1100	
3 or more	439	
*Age of youngest child (years)*	4.95	3.72

**Table 2.** Descriptive statistics for the respondents’ parents (grandparents), frequency (N) or mean (M) and standard deviation (SD).

	MGF	MGM	PGF	PGM	SMGF	SMGM	SPGF	SPGM
	*N*/*M* (±SD)	*N*/*M* (±SD)	*N*/*M* (±SD)	*N*/*M* (±SD)	*N*/*M* (±SD)	*N*/*M* (±SD)	*N*/*M* (±SD)	*N*/*M* (±SD)
Number of observations	1239	1524	662	813	218	106	110	52
*Financial support*								
Never	393	427	177	208	102	69	54	27
Sometimes	630	798	367	450	89	33	41	21
Often	216	299	118	155	27	4	15	4
*Help in taking care of children*								
Never	428	361	235	214	121	67	55	36
Sometimes	483	581	290	355	65	28	37	14
Often	328	583	137	244	32	11	18	2
*Grandparent cohabitation status*								
Not cohabiting	172	428	73	207	28	12	15	10
Cohabiting	1067	1096	589	606	190	94	95	42
*Travelling time between parent and grandparent*								
Live in the same house	87	136	81	98	10	1	7	3
Less than 10 minutes	354	455	205	258	46	13	29	6
10–30 minutes	273	342	136	173	61	39	27	10
30–60 minutes	129	144	55	73	30	12	11	10
1–3 hours	145	155	68	74	30	17	14	10
3 hours or more	251	292	117	137	41	24	22	13
*Childhood co-residence duration with parent*	17.0 (±3.33)	17.6(±2.03)	17.1(±3.2	17.6(±2.12)	2.4(±4.7)	1.0(±3.2)	2.1(±4.9)	0.23(±1.0)

MGF, maternal grandfather; MGM, maternal grandmother; PGF, paternal grandfather; PGM, paternal grandmother; MSGF, maternal step-grandfather; MSGM, maternal step-grandmother; PSGF, paternal step-grandfather; PSGM, paternal step-grandmother.

### Data analysis

Response variables were ordered in nature. However, as the proportionality assumption was violated, i.e. assumption that the distance between each outcome category is proportionate, we used generalised ordered logit/partial proportional odds model, which selectively relaxes the assumptions of the ordered logit model only as needed (Williams, 2006, 2016). In Tables 3–8 the results from proportional odds models are presented as a series of cumulative logit models; the original ordinal variable is collapsed into two categories and a series of binary logistic regressions are run. First it is category never vs. categories sometimes and often, and then never and sometimes vs. often. In each dichotomisation the lower values are recoded to 0, while the higher values are recoded to 1. A positive coefficient means that increases in the explanatory variable lead to higher levels of support or help while negative coefficients mean that increases in the explanatory value lead to less support or help (Williams, 2016). If the explanatory variable meets the proportional odds assumption, only one set of coefficients is presented. As the data are clustered within individuals, the standard errors of the estimates were corrected for clustering within individuals. To help interpret and visualise the results, we calculated the predictive margins from the regression models (Williams, 2012). All statistical analyses were performed using Stata 18 (StataCorp, 2023).

**Table 3.** Partial proportional odds model results on financial support to grandchildren (*N* = 4724). Only one set of coefficients is presented for explanatory variables that meet the proportional odds assumption

		Never vs. sometimes and often		Never and sometimes vs. often	
Explanatory variables	*p*-Value	Coefficient	Standard error	Coefficient	Standard error
Grandparent (MGM)					
MGF	<0.0001	−0.22	0.04		
PGF	0.17	−0.13	0.09		
PGM	0.99	0.001	0.09		
SMGF	<0.0001	−0.83	0.14		
SMGM	<0.0001	−1.58	0.21		
SPGF	<0.0001	−0.95	0.20	−0.36	0.29
SPGM	<0.0001	−1.02	0.28		
Cohort (1981–1983)					
1971–1973	0.93	0.01	0.11	−0.41	0.12
Ethnicity (German)					
Other countries	0.29	0.10	0.10		
Education (primary)					
Upper secondary	0.42	0.11	0.14		
Post secondary	0.15	0.25	0.17		
Tertiary	0.02	0.38	0.16		
Travel time to grandparent (same house)					
Less than 10 minutes	0.94	0.01	0.14		
10–30 minutes	0.14	−0.21	0.14		
30–60 minutes	0.24	−0.20	0.17		
1–3 hours	0.04	−0.34	0.17		
3 hours or more	<0.0001	−0.83	0.16		
Cohabitation (no)					
Cohabit with partner	0.32	−0.13	0.13		
Grandparents cohabiting (no)					
Grandparents cohabit	<0.0001	0.34	0.08		
Number of children (1)					
2	0.25	−0.10	0.09		
3 or more	<0.0001	−0.60	0.12		
Age of youngest child	<0.0001	−0.04	0.01		

First, we investigated how much financial support and help in childcare was received from grandparents by lineage. Second, we tested if childhood co-residence duration was associated with increased step-grandparental investment. We included both step-grandmothers and step-grandfathers, although we are not comparing the step-grandmothers and step-grandfathers because of the different sampling procedure. As our main variable we introduced childhood co-residence duration into the model. However, for step-grandmothers only 15 out of 158 observations of childhood co-residence existed (9.5%), whereas for grandfathers 86 out of 328 observations had childhood co-residence years (26%). For this model we did not split grandparent by lineage; instead we included the parent's sex in the model. Childhood co-residence duration with biological parents was investigated with those grandparents that were separated (were not living together with the other biological grandparent at the time of the interview). This sample included 455 grandmothers and 372 grandfathers. Childhood co-residence duration with a parent among these grandparents was on average (mean ± SD) 17 ± 3.0 years for grandmothers and 14.5 ± 5.6 for grandfathers. The mother's higher likelihood of living with underage children after divorce is evident from the data: 92 (20%) of grandmothers had less than 18 years of childhood co-residence duration, whereas for grandfathers 150 (40%) had less than 18 years of co-residence during the parent's childhood.

## Results

### Comparison of grandparental investment by different grandparent types

**Figure 1.**
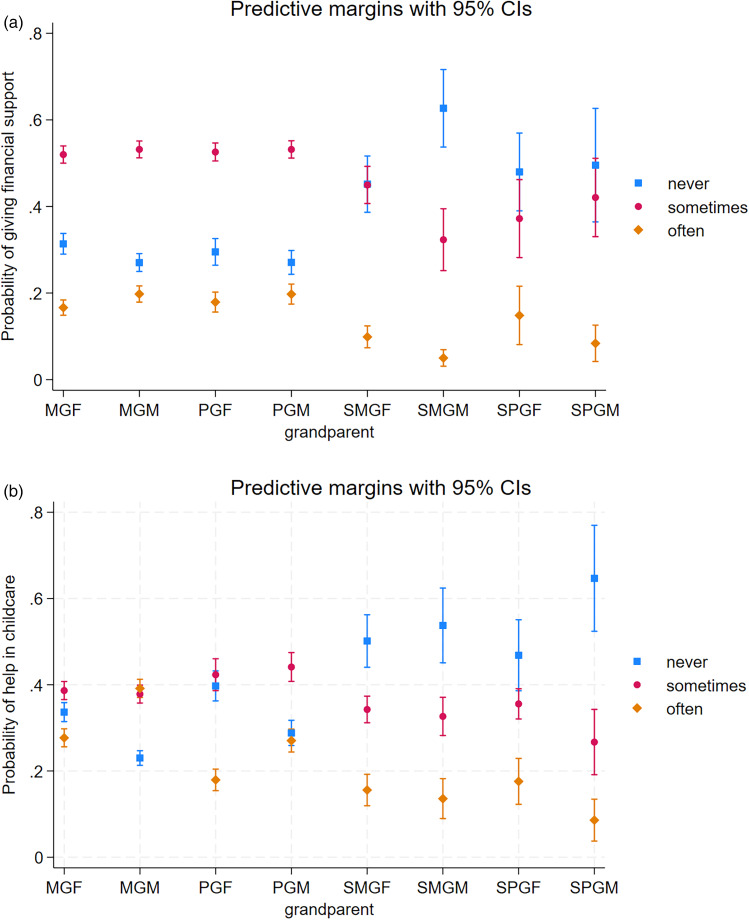
Predictive probabilities and 95% confidence intervals for giving (a) financial help and (b) help in childcare to grandchildren: never, sometimes, or often. Different (step)grandparents by lineage are shortened: MGF, maternal grandfather; MGM, maternal grandmother; PGF, paternal grandfather; PGM, paternal grandmother; SMGF, maternal step-grandfather; SMGM, maternal step-grandmother; SPGF, paternal step-grandfather; and SPGM, paternal step-grandmother.

To test Hypothesis 1 and explore whether biological grandparents invest more than step-grandparents, we first investigated the financial support given by (step)grandparents, which parents provided in response to the question ‘During the past 12 months, did you receive from (step)parent substantial gifts or financial support for your children?’ Results indicate that other grandparents except the paternal grandmother and paternal grandfather were less likely to give financial support than the maternal grandmother. Overall, Figure 1 illustrates a higher probability of giving financial support sometimes or often by biological grandparents than step-grandparents, whereas step-grandparents were more likely to never give financial support (Table 3; Figure 1a). Unadjusted results are found in supplementary Table S1.

Maternal vs. paternal lineage differences were not evident between grandfathers, step-grandfathers or step-grandmothers. Having three or more biological children, a travelling time of more than one hour to grandparents and older age of youngest child were associated with less support from grandparents. Positive association was found with grandparental cohabitation and tertiary education of parents.

The second measure of grandparental investment given by (step)grandparents was help in childcare, which was provided by parents in response to the question: ‘During the past 12 months, how often did you receive help from (step)parent in looking after or taking care of your children?’ Maternal grandmothers gave more help in childcare than any other grandparent type; maternal grandmothers were most likely to give childcare often and least likely never to help in childcare (Table 4; Figure 1b). Maternal grandfathers were more likely to help in childcare often compared with paternal grandfathers. Paternal grandfathers were as likely to never help than help sometimes, whereas paternal grandmothers were as likely to help often than never, but most likely to help sometimes (Figure 1b). Lineage differences were not present between step-fathers or step-mothers; overall step-grandparents were most likely to be never helping in childcare. More educated parents received more help in childcare; however, in families with three or more children and where the youngest child is older, less childcare was received. Longer travelling time to grandparents’ house was associated with less help in childcare and cohabiting grandparents gave more help in childcare. Unadjusted results are in line with adjusted ones (supplementary Table 2).

**Table 4.** Partial proportional odds model results on help in childcare of grandchildren (*N* = 4724). Only one set of coefficients is presented for explanatory variables that meet the proportional odds assumption

		Never vs. sometimes and often		Never and sometimes vs. often	
Explanatory variables	*p*-Value	Coefficient	Standard error	Coefficient	Standard error
Grandparent (MGM)					
MGF	<0.0001	−0.66	0.05		
PGF	<0.0001	−0.99	0.11	−1.33	0.12
PGM	<0.01	−0.38	0.11	−0.70	0.10
SMGF	<0.0001	−1.53	0.15		
SMGM	<0.0001	−1.71	0.23		
SPGF	<0.0001	−1.36	0.22		
SPGM	<0.0001	−2.28	0.34		
Cohort (1981–1983)					
1971–1973	0.96	−0.005	0.10		
Ethnicity (German)					
Other countries	0.10	−0.17	0.11	0.14	0.11
Education (primary)					
Upper secondary	<0.01	0.48	0.14		
Post secondary	<0.0001	0.76	0.18		
Tertiary		1.26	0.17	0.87	0.17
Travel time to grandparent (same house)					
Less than 10 minutes	<0.0001	−0.90	0.15		
10–30 minutes	<0.0001	−1.57	0.16		
30–60 minutes	<0.0001	−2.15	0.18		
1–3 hours	<0.0001	−2.75	0.19	-3.21	0.25
3 hours or more	<0.0001	−3.23	0.18	−3.81	0.26
Cohabitation (no)					
Cohabit with partner	0.95	0.01	0.12		
Grandparents cohabiting (no)					
Grandparents cohabit	<0.0001	0.42	0.08		
Number of children (1)					
2	0.2	−0.11	0.09		
3 or more	<0.01	−0.35	0.12		
Age of youngest child	<0.0001	−0.06	0.01		

### Childhood co-residence duration and step-grandparental investment

**Figure 2.**
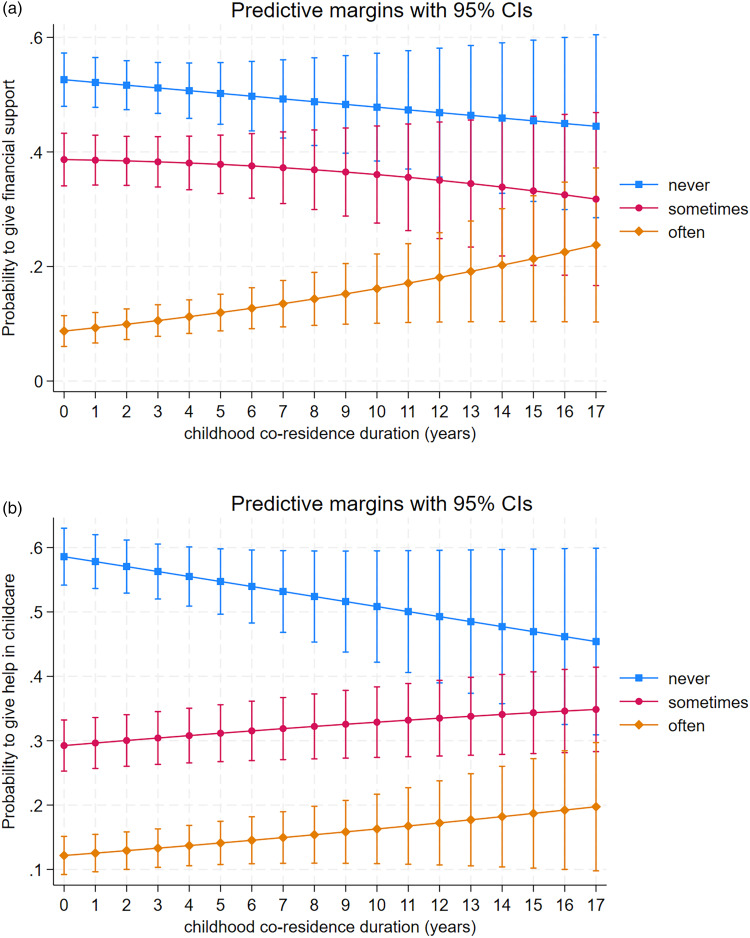
Predictive probabilities and 95% confidence intervals for step-grandparents to give (a) financial help and (b) help in childcare never, sometimes, or often in relation to childhood co-residence duration with parent of grandchildren in years.

To test Hypothesis 2, we explored whether childhood co-residence duration with step-grandparents was associated with the amount of financial support and help with childcare given. The model for giving financial support suggested that the effect of childhood co-residence duration was not proportional and, model predicted probabilities suggested a slight positive association of giving financial support often with increasing childhood co-residence duration. However, high variation does not support a strong association (Table 5, Figure 2a). The probability of helping in childcare was not associated with childhood co-residence duration (Table 6, Figure 2b).

**Table 5.** Results from partial proportional odds model investigating the relationship between financial support of step-grandmothers and step-grandfathers and childhood co-residence (*N* = 486). Only one set of coefficients is presented for explanatory variables that meet the proportional odds assumption

		Never vs. sometimes and often		Never and sometimes vs. often	
Explanatory variables	*p*-Value	Coefficient	Standard error	Coefficient	Standard error
Step-grandparent (step-grandfather)					
Step-grandmother	0.03	−0.45	0.21		
Sex (male)					
Female	0.32	−0.20	0.20		
Childhood co-residence duration	0.36	0.02	0.02	0.07	0.03
Cohort (1981–1983)					
1971–1973	0.57	−0.13	0.24		
Ethnicity (German)					
Other countries	0.75	0.09	0.27		
Education (primary)					
Upper secondary	0.80	0.08	0.32		
Post secondary	0.77	0.12	0.40		
Tertiary	0.75	0.12	0.36		
Travel time to grandparent (same house)					
Less than 10 minutes	0.64	−0.22	0.47		
10–30 minutes	0.14	−0.67	0.46		
30–60 minutes	0.65	−0.23	0.49		
1–3 hours	0.58	−0.27	0.49		
3 hours or more	0.01	−1.33	0.49		
Cohabitation (no)					
Cohabit with partner	0.50	−0.18	0.26		
Grandparents cohabiting (no)					
Grandparents cohabit	0.02	0.65	0.29		
Number of children (1)					
2	0.86	0.04	0.21		
3 or more	0.10	−0.47	0.29		
Age of youngest child	0.35	−0.03	0.03

**Table 6.** Results from partial proportional odds model investigating the relationship between help in childcare of step-grandmothers and step-grandfathers and childhood co-residence (*N* = 486). Only one set of coefficients is presented for explanatory variables, because the proportional odds assumption was met for all explanatory variables

Explanatory variables	*p*-Value	Coefficient	Standard error
Step-grandparent (step-grandfather)			
Step-grandmother	0.12	−0.33	0.21
Sex (male)			
Female	0.65	−0.10	0.22
Childhood co-residence duration	0.11	0.04	0.02
Cohort (1981–1983)			
1971–1973	0.48	−0.18	0.26
Ethnicity (German)			
Other countries	0.65	−0.12	0.27
Education (primary)			
Upper secondary	0.91	−0.04	0.37
Post secondary	0.83	0.09	0.43
Tertiary	0.66	0.18	0.40
Travel time to grandparent (same house)			
Less than 10 minutes	0.13	−0.62	0.40
10–30 minutes	0.02	−0.94	0.39
30–60 minutes	<0.01	−1.30	0.45
1–3 hours	<0.0001	−1.90	0.43
3 hours or more	<0.0001	−2.63	0.44
Cohabitation (no)			
Cohabit with partner	0.99	0.01	0.32
Grandparents cohabiting (no)			
Grandparents cohabit	0.14	0.42	0.28
Number of children (1)			
2	0.54	−0.14	0.23
3 or more	0.94	−0.02	0.30
Age of youngest child	0.33	−0.03	0.03

### Co-residence duration and investment of separated grandparents

**Figure 3.**
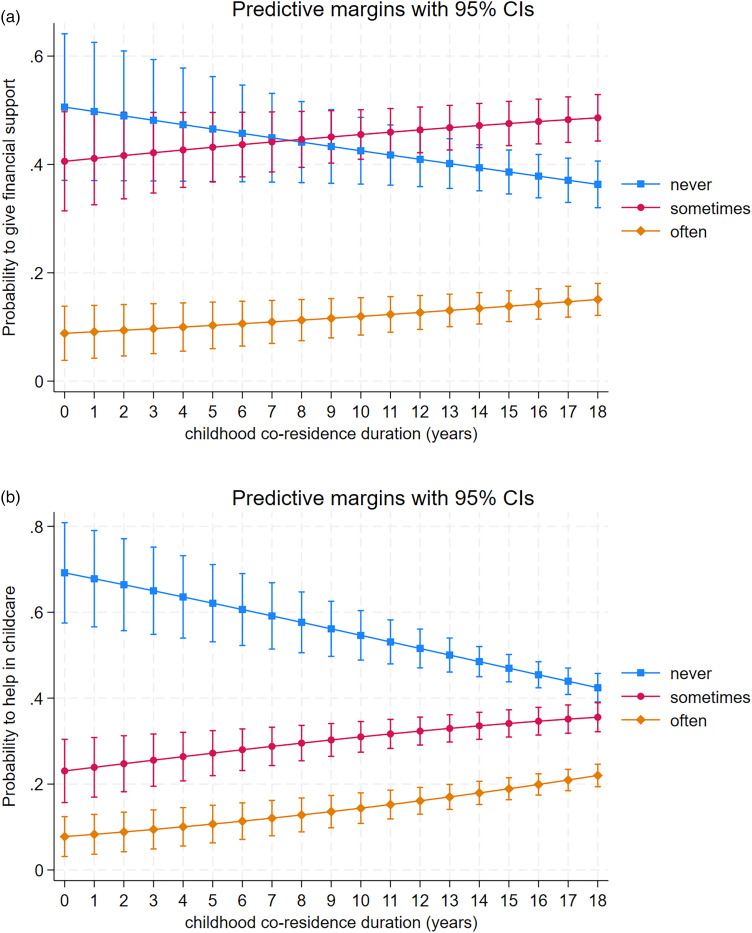
Predictive probabilities and 95% confidence intervals for separated biological grandparents to give (a) financial help and (b) help in childcare never, sometimes, or often in relation to childhood co-residence duration with parent of grandchildren in years.

To test Hypothesis 3, we investigated the relationship between biological grandparents’ co-residence with parents and the financial support and help in childcare they received. Here, the analyses were restricted to biological grandparents that had separated (i.e. grandmother and grandfather did not live together), because childhood co-residence duration of parents and grandparents would be almost exclusively 18 years for those biological grandparents who were still together at the time of the survey. Childhood co-residence duration was weakly positively associated with financial support (Table 7 and Figure 3a). For help in childcare, there was a positive association with longer co-residence with the parent of the grandchildren that associated with a higher probability of giving help in childcare often or sometimes, whereas the association was negative for never helping with childcare (Table 8 and Figure 3b). Daughters were especially more likely to report getting help in childcare *often* compared with sons (predictive margin ± SE: 0.24 ± 0.02 and 0.13 ± 0.02, respectively).

**Table 7.** Results from partial proportional odds model investigating the relationship between financial support of separated grandmothers and grandfathers and childhood co-residence (*N =* 827). Only one set of coefficients is presented for explanatory variables that meet the proportional odds assumption

		Never vs. sometimes and often		Never and sometimes vs. often	
Explanatory variables	*p*-Value	Coefficient	Standard error	Coefficient	Standard error
Grandparent (grandmother)					
Grandfather	<0.01	−0.45	0.14		
Sex (male)					
Female	0.06	−0.29	0.15		
Childhood co-residence duration	0.04	0.03	0.02		
Cohort (1981–1983)					
1971–1973	0.24	−0.21	0.18		
Ethnicity (German)					
Other countries	0.36	0.16	0.18		
Education (primary)					
Upper secondary	0.66	0.09	0.22		
Post secondary	0.41	0.23	0.28		
Tertiary	0.05	0.54	0.27	−0.08	0.32
Travel time to grandparent (same house)					
Less than 10 minutes	0.13	0.45	0.30		
10–30 minutes	0.32	0.30	0.30		
30–60 minutes	<0.01	0.93	0.33		
1–3 hours	0.18	0.46	0.34		
3 hours or more	0.57	−0.17	0.30		
Cohabitation (no)					
Cohabit with partner	0.58	−0.10	0.18		
Grandparents cohabiting (no)					
Grandparents cohabit	0.39	−0.12	0.14		
Number of children (1)					
2	0.77	−0.05	0.15		
3 or more	<0.0001	−0.77	0.21		
Age of youngest child	0.59	0.01	0.02

**Table 8.** Results from partial proportional odds model investigating the relationship between help in childcare of separated grandmothers and grandfathers and childhood co-residence (*N =* 827). Only one set of coefficients is presented for explanatory variables that meet the proportional odds assumption

		Never vs. sometimes and often		Never and sometimes vs. often	
Explanatory variables	*p*-Value	Coefficient	Standard error	Coefficient	Standard error
Grandparent (grandmother)					
Grandfather	<0.0001	−1.21	0.15		
Sex (male)					
Female	0.06	0.34	0.18	0.94	0.23
Childhood co-residence duration	<0.0001	0.08	0.02		
Cohort (1981–1983)					
1971–1973	0.97	0.01	0.19		
Ethnicity (German)					
Other countries	0.15	0.28	0.19		
Education (primary)					
Upper secondary	0.03	0.52	0.24		
Post secondary	0.46	0.23	0.31		
Tertiary	0.01	0.78	0.28		
Travel time to grandparent (same house)					
Less than 10 minutes	0.02	−0.72	0.31		
10–30 minutes	<0.0001	−1.14	0.31		
30–60 minutes	<0.0001	−1.41	0.34		
1–3 hours	<0.0001	−2.34	0.37		
3 hours or more	<0.0001	−2.62	0.34	−3.49	0.50
Cohabitation (no)					
Cohabit with partner	0.65	0.09	0.19		
Grandparents cohabiting (no)					
Grandparents cohabit	0.03	−0.32	0.15		
Number of children (1)					
2	0.54	−0.10	0.16		
3 or more	<0.01	−0.63	0.23		
Age of youngest child	0.05	−0.05	0.02		

## Discussion

Our analysis provides evidence that step-grandparents offer comparatively lower levels of financial support and assistance in childcare when contrasted with biological grandparents. This finding aligns with previous research that has consistently shown that step-grandparents invest less in their grandchildren compared with biological grandparents (e.g. Coall et al., 2014; Daly & Perry, 2021; Pashos et al., 2016). However, diverging from earlier findings suggesting that step-grandfathers invest more than biological grandfathers (Gray & Brogdon, 2017; van Houdt et al., 2019), our study revealed that biological grandfathers contribute more to their grandchildren than step-grandfathers do. Importantly, the studies by Gray and Brogdon (2017) and van Houdt and colleagues (2019) reporting higher investment by step-grandfathers were conducted using within-subject analyses, involving samples that encompass individuals with both types of grandfathers (Gray & Brogdon, 2017; van Houdt et al., 2019). However, these estimates omit those grandfathers who have not separated from the grandmother and are investing more than separated grandfathers (Daly & Perry, 2021; Danielsbacka & Tanskanen, 2018). Therefore, it is important to considerer whether step-grandparents are compared with separated biological grandparents or all biological grandparents.

We found that grandmothers, especially maternal grandmothers, were more likely to give help in childcare. Overall, there exists a notable sex-based discrepancy in the level of involvement with grandchildren. Grandmothers are typically expected to provide more childcare (Horsfall & Dempsey, 2015), and indeed they do spend more time caring for their grandchildren in comparison with grandfathers (Di Gessa et al., 2020). It has been proposed that owing to the stronger involvement of grandmothers with their grandchildren, the extent of grandfathers’ investment in their grandchildren is more reliant on having a grandmother as a spouse (Euler, 2011; Pollet et al., 2006). For instance, in Finland, divorce and remarriage were associated with reduced childcare and decreased contact with grandchildren among grandfathers, whereas among grandmothers, only remarriage was associated with lower levels of contact and childcare (Danielsbacka & Tanskanen, 2018). Furthermore, Daly and Perry (2021) in their examination of European grandparents revealed that grandfathers tend to direct their investment predominantly toward the grandchildren of their current partners, potentially at the expense of grandchildren from previous relationships. In contrast, women appear to prioritise their own biological grandchildren. This is in line with the grandmother hypothesis, which posits that the evolution of extended post-menopausal lifespan in women is driven by natural selection because women can aid their own offspring in reproduction and care (Hawkes et al., 1998).

We further explored whether the duration of childhood co-residence could be linked to step-grandparental and grandparental investment. The time spent together during childhood has the potential to foster stronger bonds between parents and children that can have effects on intergenerational relationships in later life. The age of the child at the onset of step parenthood is also acknowledged as a potential factor influencing step-relationships (e.g. Hornstra et al., 2020; Kalmijn, 2013; Pettay et al., 2023). In the present study, we were unable to identify a connection between childhood co-residence and step-grandparental investment. However, a positive association between co-residence duration and grandparental investment was observed among biological grandfathers who were separated from the biological grandmothers. This discrepancy could suggest that the kin-like closeness associated with co-residence might not extend to step-grandchildren, whereas with biological grandparents, shared residence with the parent could translate potentially through increased attachment relationships to higher levels of grandparental investment. These findings also hint at the possibility that step-grandparental investment might primarily stem from motivations related to mating effort. While persons who become grandparents are typically not reproductive themselves, pair bonding has many advantages, and marital status is known to be related to increased survival in many studies (see Manzoli et al., 2007 for review). Pair bonding might be especially important for the wellbeing of men at older ages. For example, men suffered more in terms of psychological wellbeing than women following the loss of a spouse in a Canadian population, and gaining a spouse had positive consequences for men's life satisfaction but not for women's (Chipperfield & Havens, 2001). Overall, the spouse is often the most important person for social support, especially for men (Shenk et al., 2013).

An alternative explanation of mating effort for the investment of grandfathers may be a product of recent demographic changes influencing family structures. With increasing rates of divorce and the higher rates of re-marriage in males, grandfathers may be in an emerging niche where they are more likely to be available to support step-children and step-grandchildren and provide replacement or additional parenting resources (Coall et al., 2016). In these step-family environments, several factors contribute to a potential increased role for step-grandfathers: grandfathers that are more likely to be retired; second parent figures (often fathers) that are more likely to be absent; increasing costs and decreased availability of formal childcare; increased reliance on kin for childcare; and the ever increasing demand for more investment in children (Coall et al., 2016). While we cannot explore this possibility with this data, future studies should address the changing roles of (step)grandfathers.

While the current study focused on step-grandparents who themselves are step-parents of parents, another route to step-grandparenthood is when a biological child becomes a step-parent. Contact with grandparents and relationships with grandparents in divorced families has gained attention as grandparents can provide additional support in times of need (Anderson et al., 2018; Coall et al., 2018). Remarriage of parents can also bring step-grandparents into a child's life. Lussier and colleagues (2002) found that in an English population, contact with step-parents’ parents was similar to that of a biological grandparent if the step-parent was living with the child. However, some research suggests that less grandparental investment is given to step-families, which indirectly suggests grandparental discrimination against step-grandchildren (Tanskanen et al., 2014, 2020).

Our study has some limitations. Cross-sectional data do not allow us to draw definite causal conclusions. One of the limitations of this study is that we cannot be sure that the last childhood co-residency before age of 18 with a step-parent is the same step-parent as at the time of the survey. These data also prioritised step-fathers, and questions about step-mothers were asked only when step-fathers did not exist. This limits our conclusions about step-grandmothers. Owing to the lack of data points on step-grandmothers or mothers with less than full childhood co-residence duration, the patterns between childhood co-residence duration and investment are probably driven by step-grandfathers and separated grandfathers. This lack of variation in childhood co-residence duration with (step)grandmothers means that we cannot investigate whether the relationship between co-residence duration and investment would be sex-specific. Furthermore, future research could benefit from considering the presence of other grandchildren and step-grandchildren, which may affect the provision of intergenerational support (Daly & Perry 2021). A lack of information on grandparents’ income and health is also a limitation of our analyses. Finally, our results are based on a sample of German people, which may limit the generalisability of the findings to other countries, especially outside WEIRD (Western, educated, industrialised, rich, democratic) countries (Henrich et al., 2010).

Our study suggests that step-grandparents give less help in childcare and financial support compared with biological grandparents. Length of childhood co-residence with the separated biological parent of a grandchild was associated with increased investment, while for step-grandparents childhood co-residence with the parent did not relate to the investment. Investment from step-grandparents might thus be mainly interpreted as mating effort. However, childhood co-residence with parents boosted biological grandparents’ investment, suggesting that biological relatedness is not the sole contributor to grandparental investment. In aging populations, with recent demographic changes, the number of step-grandparents in grandchildren's lives are likely to be increasing; therefore, we need further detailed investigations of the roles that step-grandparents play in families.

## Supporting information

Pettay et al. supplementary material 1 Pettay et al. supplementary material

Pettay et al. supplementary material 2 Pettay et al. supplementary material

## Supplementary material

To view supplementary material for this article, please visit https://doi.org/10.1017/ehs.2024.17

## Research transparency and reproducibility

The data that support the findings of this study were provided by the German Family Panel. The data are available at https://www.pairfam.de/en/data/data-access/ for scholarly analysis. Stata code used to conduct statistical analysis can be found in Supporting Information 2.
