# Supplementary material for: The role of mating effort and co-residence history in step-grandparental investment
Source: Evol Hum Sci. 2024 May 16;6:e27. doi: 10.1017/ehs.2024.17 (PMC11106544; doi:10.1017/ehs.2024.17)
Supplement: Pettay et al. supplementary material 1 — Pettay et al. supplementary material [file S2513843X24000173sup001.pdf]

Online supplementary tables for:

The role of mating effort and co-residence history in step-grandparental investment

Jenni E. Pettay, David A. Coall, Mirkka Danielsbacka and Antti O. Tanskanen

Supplementary table 1. Partial proportional odds model results on financial support to grandchildren with only grandparental type as explanatory variable (N =4,724). All variables fulfilled parallel-lines assumption.

Explanatory variables	p value	coefficient	std.err
grandparent (MGM)			
MGF	<.0001	-0.17	0.04
PGF	0.91	-0.01	0.09
PGM	0.49	0.06	0.08
SMGF	<.0001	-0.77	0.13
SMGM	<.0001	-1.59	0.20
SPGF	<.0001	-0.82	0.21
SPGM	<.0001	-1.04	0.27

Supplementary table 2. Partial proportional odds model results for help in childcare with only grandparental type as explanatory variable (N =4,724). Only one set of coefficients is presented for explanatory variables that meet the proportional odds assumption.

		never vs. sometimes & often		never & sometimes vs. often	
Explanatory variables	p value	coefficient	std.err	coefficient	std.err
grandparent (MGM)					
MGF	<.0001	-0.54	0.05		
PGF	<.0001	-0.57	0.10	-0.86	0.11
PGM	0.14	-0.14	0.10	-0.37	0.09
SMGF	<.0001	-1.37	0.13		
SMGM	<.0001	-1.71	0.20		
SPGF	<.0001	-1.17	0.19		
SPGM	<.0001	-2.04	0.28		

Supplementary table 3. Partial proportional odds model results on financial support to grandchildren including design weight (N =4,724). Only one set of coefficients is presented for explanatory variables that meet the proportional odds assumption.

		never vs. sometimes & often		never & sometimes vs. often	
Explanatory variables	p value	coefficient	std.err	coefficient	std.err
grandparent (MGM)					
MGF	<.0001	-0.21	0.04		
PGF	0.18	-0.12	0.09		
PGM	1.00	0.001	0.09		
SMGF	<.0001	-0.83	0.14		
SMGM	<.0001	-1.58	0.21		
SPGF	<.0001	-0.95	0.20	-0.36	0.29
SPGM	<.0001	-1.00	0.28		
cohort (1981-1983)					
1971-1973	0.93	0.01	0.11	-0.41	0.12
ethnicity (German)					
other countries	0.34	0.09	0.10		
education (primary)					
upper secondary	0.45	0.11	0.14		
post secondary	0.15	0.25	0.17		
tertiary	0.02	0.37	0.16		
travel time to GP (same house)					
Less than 10 minutes	0.99	0.00	0.14		
10-30 minutes	0.11	-0.23	0.14		
30-60 minutes	0.22	-0.21	0.17		
1-3 hours	0.03	-0.36	0.17		
3 hours or more	<.0001	-0.83	0.16		
cohabitation (no)					
cohabit with partner	0.29	-0.13	0.13		
grandparents cohabiting (no)					
grandparents cohabit	<.0001	0.34	0.08		
number of children (1)					
2	0.23	-0.11	0.09		
3 or more	<.0001	-0.59	0.12		
age of youngest child	<.01	-0.04	0.01		

Supplementary table 4. Partial proportional odds model results on help in childcare of grandchildren including design weight (N =4,724). Only one set of coefficients is presented for explanatory variables that meet the proportional odds assumption.

		never vs. sometimes & often		never & sometimes vs. often	
Explanatory variables	p value	coefficient	std.err	coefficient	std.err
grandparent (MGM)					
MGF	<.0001	-0.65	0.05		
PGF	<.0001	-0.99	0.11	-1.33	0.12
PGM	<.0001	-0.38	0.11	-0.71	0.10
SMGF	<.0001	-1.53	0.15		
SMGM	<.0001	-1.72	0.23		
SPGF	<.0001	-1.37	0.22		
SPGM	<.0001	-2.28	0.35		
cohort (1981-1983)					
1971-1973	0.97	0.00	0.10		
ethnicity (German)					
other countries	0.08	-0.18	0.11	0.13	0.11
education (primary)					
upper secondary	<.01	0.48	0.14		
post secondary	<.0001	0.76	0.18		
tertiary	<.0001	1.26	0.17	0.87	0.17
travel time to GP (same house)					
Less than 10 minutes	<.0001	-0.89	0.15		
10-30 minutes	<.0001	-1.56	0.16		
30-60 minutes	<.0001	-2.15	0.18		
1-3 hours	<.0001	-2.75	0.19	-3.21	0.25
3 hours or more	<.0001	-3.21	0.18	-3.79	0.26
cohabitation (no)					
cohabit with partner	0.95	0.01	0.12		
grandparents cohabiting (no)					
grandparents cohabit	<.0001	0.42	0.08		
number of children (1)					
2	0.20	-0.11	0.09		
3 or more	<.01	-0.35	0.12		
age of youngest child	<.0001	-0.07	0.01		

Supplementary table 5. Results from partial proportional odds model investigating the relationship between financial support of step-grandmothers and step-grandfathers and childhood co-residence including weight (N=486). Only one set of coefficients is presented for explanatory variables that meet the proportional odds assumption

		never vs. sometimes & often		never & sometimes vs. often	
Explanatory variables	p value	coefficient	std.err	coefficient	std.err
step-grandparent (step-grandfather)					
step-grandmother	0.02	-0.45	0.20		
sex (male)					
female	0.33	-0.21	0.21		
childhood co-residence duration	0.36	0.02	0.02	0.07	0.03
cohort (1981-1983)					
1971-1973	0.59	-0.13	0.25		
ethnicity (German)					
other countries	0.80	0.07	0.28		
education (primary)	0.83	0.07	0.34		
upper secondary	0.79	0.11	0.40		
post secondary	0.78	0.11	0.38		
tertiary					
travel time to GP (same house)					
Less than 10 minutes	0.63	-0.23	0.48		
10-30 minutes	0.14	-0.69	0.47		
30-60 minutes	0.62	-0.25	0.50		
1-3 hours	0.56	-0.29	0.51		
3 hours or more	0.01	-1.32	0.51		
cohabitation (no)					
cohabit with partner	0.43	-0.21	0.27		
grandparents cohabiting (no)					
grandparents cohabit	0.02	0.65	0.29		
number of children (1)					
2	0.84	0.04	0.21		
3 or more	0.11	-0.47	0.30		
age of youngest child	0.35	-0.03	0.03		

Supplementary table 6. Results from partial proportional odds model investigating the relationship between help in childcare of step-grandmothers and step-grandfathers and childhood co-residence including design weight (N=486). Only one set of coefficients is presented for explanatory variables, because the proportional odds assumption was met for all explanatory variables.

Explanatory variables	p value	coefficient	std.err
step-grandparent (step-grandfather)			
step-grandmother	0.12	-0.33	0.21
sex (male)			
female	0.65	-0.09	0.21
childhood co-residence duration	0.10	0.04	0.02
cohort (1981-1983)			
1971-1973	0.47	-0.18	0.25
ethnicity (German)			
other countries	0.74	-0.10	0.28
education (primary)			
upper secondary	0.93	-0.03	0.33
post secondary	0.82	0.09	0.41
tertiary	0.68	0.15	0.38
travel time to GP (same house)			
Less than 10 minutes	0.14	-0.66	0.45
10-30 minutes	0.03	-0.93	0.44
30-60 minutes	0.01	-1.29	0.48
1-3 hours	<.001	-1.90	0.49
3 hours or more	<.001	-2.64	0.49
cohabitation (no)			
cohabit with partner	0.96	0.02	0.28
grandparents cohabiting (no)			
grandparents cohabit	0.15	0.42	0.29
number of children (1)			
2	0.52	-0.14	0.22
3 or more	0.92	-0.03	0.29
age of youngest child	0.33	-0.03	0.03

Supplementary table 7. Results from partial proportional odds model investigating the relationship between financial support of separated grandmothers and grandfathers and childhood co-residence including design weight (N=827). Only one set of coefficients is presented for explanatory variables that meet the proportional odds assumption.

		never vs. sometimes & often		never & sometimes vs. often	
Explanatory variables	p value	coefficient	std.err	coefficient	std.err
grandparent (grandmother)					
grandfather	<.01	-0.43	0.14		
sex (male)					
female	0.07	-0.29	0.16		
childhood co-residence duration	0.05	0.03	0.02		
cohort (1981-1983)					
1971-1973	0.23	-0.21	0.18		
ethnicity (German)					
other countries	0.40	0.16	0.19		
education (primary)					
upper secondary	0.69	0.09	0.22		
post secondary	0.40	0.23	0.27		
tertiary	0.05	0.54	0.27	-0.10	0.32
travel time to GP (same house)					
Less than 10 minutes	0.11	0.48	0.30		
10-30 minutes	0.32	0.31	0.31		
30-60 minutes	0.01	0.93	0.33		
1-3 hours	0.19	0.47	0.36		
3 hours or more	0.61	-0.16	0.31		
cohabitation (no)					
cohabit with partner	0.61	-0.09	0.18		
grandparents cohabiting (no)					
grandparents cohabit	0.38	-0.13	0.14		
number of children (1)					
2	0.81	-0.04	0.16		
3 or more	<.0001	-0.76	0.21		
age of youngest child	0.60	0.01	0.02		

Supplementary table 8. Results from partial proportional odds model investigating the relationship between help in childcare of separated grandmothers and grandfathers and childhood co-residence including design weight (N=827). Only one set of coefficients is presented for explanatory variables that meet the proportional odds assumption.

		never vs. sometimes & often		never & sometimes vs. often	
Explanatory variables	p value	coefficient	std.err	coefficient	std.err
grandparent (grandmother)					
grandfather	<.0001	-1.19	0.16		
sex (male)					
female	0.073	0.33	0.19	1.00	0.24
childhood co-residence duration	<.0001	0.08	0.02		
cohort (1981-1983)					
1971-1973	0.96	0.01	0.19		
ethnicity (German)					
other countries	0.16	0.27	0.19		
education (primary)					
upper secondary	0.07	0.48	0.27		
post secondary	0.28	-0.32	0.41	-0.32	0.41
tertiary	0.02	-2.58	0.36		
travel time to GP (same house)					
Less than 10 minutes	0.0410	-0.68	0.33		
10-30 minutes	<.01	-1.10	0.34		
30-60 minutes	<.0001	-1.38	0.36		
1-3 hours	<.0001	-2.31	0.38		
3 hours or more	<.0001	-2.58	0.36	-3.44	0.51
cohabitation (no)					
cohabit with partner	0.57	0.11	0.19		
grandparents cohabiting (no)					
grandparents cohabit	0.04	-0.31	0.15		
number of children (1)					
2	0.55	-0.10	0.16		
3 or more	<.01	-0.64	0.22		
age of youngest child	0.05	-0.05	0.02		
