# Supplementary material for: The role of mating effort and co-residence history in step-grandparental investment
Source: Evol Hum Sci. 2024 May 16;6:e27. doi: 10.1017/ehs.2024.17 (PMC11106544; doi:10.1017/ehs.2024.17)
Supplement: Pettay et al. supplementary material 2 — Pettay et al. supplementary material [file S2513843X24000173sup002.pdf]

Supplementary material 2

Stata 18.0 code for analysis in:

The role of mating effort and co-residence history in step-grandparental investment

Jenni E. Pettay, David A. Coall, Mirkka Danielsbacka and Antti O. Tanskanen

The data that support the findings of this study were provided by the German Family Panel (pairfam; https://www.pairfam.de/en/data/data-access/). Data of wave 2 was used in these analyisis.

```
use anchor2.dta

*English labels

label language en


*** see pairfam Data Manual Release 10.0 for code explanations


***Time spend with each parent or stepparent before adulthood


* cla7e1 age when family changed

* cla6ex with whom lived

* cl3 lived with both parents after birth

* cla4 with whom lived after birth

* cla5ex any changes in living composition before age 18


* with whom lived at birth and during childhood


keep id age cla3* cla4* cla5e* cla6e* cla7e*
```

```
recode cla7e1 (-4 -3 -2 -1=.)
recode cla7e2 (-4 -3 -2 -1=.)
recode cla7e3 (-4 -3 -2 -1=.)
recode cla7e4 (-4 -3 -2 -1=.)
recode cla7e5 (-4 -3 -2 -1=.)
recode cla7e6 (-4 -3 -2 -1=.)
recode cla7e7 (-4 -3 -2 -1=.)
recode cla7e8 (-4 -3 -2 -1=.)
recode cla7e9 (-4 -3 -2 -1=.)

recode cla6e1 (-4 -3 -2 -1=.)
recode cla6e2 (-4 -3 -2 -1=.)
recode cla6e3 (-4 -3 -2 -1=.)
recode cla6e4 (-4 -3 -2 -1=.)
recode cla6e5 (-4 -3 -2 -1=.)
recode cla6e6 (-4 -3 -2 -1=.)
recode cla6e7 (-4 -3 -2 -1=.)
recode cla6e8 (-4 -3 -2 -1=.)
recode cla6e9 (-4 -3 -2 -1=.)


tabulate cla3
tabulate cla4


reshape long cla6e cla7e , i(id) j(ch_change)

recode cla5e1 (-4 -3 -2 -1=.) (2 = 0) (1 = 1), gen(eroperhe)

recode cla3 (-2 -1=.) (2 = 0) (1 = 1), gen(ydinperhe)
```

```
recode cla4 (-3 -2 -1=.), gen(syntyessa)

generate ekaperhe = .
replace ekaperhe = 1 if cla3 == 1
replace ekaperhe = cla4 if cla3 != 1
replace ekaperhe = . if ekaperhe == -3

tabulate ekaperhe cla3

reshape wide cla6e cla7e , i(id) j(ch_change)

* time periods when family composition changed

generate pituus0 = .
replace pituus0 = cla7e1 if cla7e1 != .
replace pituus0 = age if age <= 18 &  cla5e1 == 2
replace pituus0 = 18 if age > 18 & cla5e1 == 2


generate pituus1 = .
replace pituus1 = cla7e2-cla7e1 if cla7e2 != . & cla7e1 != .
replace pituus1 = 18  if cla7e1 == .
replace pituus1 = age if age < 18 & cla7e1 == .
replace pituus1 = 18 - cla7e1 if cla7e2 == . & cla7e1 != .
replace pituus1 = age - cla7e1 if age < 18 & cla7e2 == . & cla7e1 != .

generate pituus2 = .
replace pituus2 = cla7e3-cla7e2 if cla7e2 != . & cla7e3 != .
replace pituus2 = 18 - cla7e2 if cla7e3 == . & cla7e2 != .
replace pituus2 = age - cla7e2 if age < 18 & cla7e3 == . & cla7e2 != .
```

```
generate pituus3 = .

replace pituus3 = cla7e4-cla7e3 if cla7e3 != . & cla7e4 != .

replace pituus3 = 18 - cla7e3 if cla7e4 == . & cla7e3 != .

replace pituus3 = age - cla7e3 if age < 18 & cla7e4 == . & cla7e3 != .


generate pituus4 = .

replace pituus4 = cla7e5-cla7e4 if cla7e4 != . & cla7e5 != .

replace pituus4 = 18 - cla7e4 if cla7e5 == . & cla7e4 != .

replace pituus4 = age - cla7e4 if age <18 & cla7e5 == . & cla7e4 != .


generate pituus5 = .

replace pituus5 = cla7e6-cla7e5 if cla7e5 != . & cla7e6 != .

replace pituus5 = 18 - cla7e5 if cla7e6 == . & cla7e5 != .

replace pituus5 = age - cla7e5 if age < 18 &cla7e6 == . & cla7e5 != .


generate pituus6 = .

replace pituus6 = cla7e7-cla7e6 if cla7e6 != . & cla7e7 != .

replace pituus6 = 18 - cla7e6 if cla7e7 == . & cla7e6 != .

replace pituus6 = age- cla7e6 if age < 18 & cla7e7 == . & cla7e6 != .


generate pituus7 = .

replace pituus7 = cla7e8-cla7e7 if cla7e7 != . & cla7e8 != .

replace pituus7 = 18 - cla7e7 if cla7e8 == . & cla7e7 != .

replace pituus7 = age - cla7e7 if age < 18 & cla7e8 == . & cla7e7 != .


generate pituus8 = .

replace pituus8 = cla7e9-cla7e8 if cla7e8 != . & cla7e9 != .

replace pituus8 = 18 - cla7e8 if cla7e9 == . & cla7e8 != .

replace pituus8 = age - cla7e8 if age < 18 & cla7e9 == . & cla7e8 != .


generate pituus9 = .
```

```
replace pituus9 = 18 - cla7e9 if cla7e9 != .

replace pituus9 = age  - cla7e9 if  age < 18 & cla7e9 != .


* childhood time with mother = M, codes 1,2,3


generate Mpituus0 = .

replace Mpituus0 = pituus0 if ekaperhe == 1 | ekaperhe == 2 | ekaperhe == 3



generate Mpituus1 = .

replace Mpituus1 = pituus1 if cla6e1 == 1 | cla6e1 == 2 | cla6e1 == 3


generate Mpituus2 = .

replace Mpituus2 = pituus2 if cla6e2 == 1 | cla6e2 == 2 | cla6e2 == 3


generate Mpituus3 = .

replace Mpituus3 = pituus3 if cla6e3 == 1 | cla6e3 == 2 | cla6e3 == 3


generate Mpituus4 = .

replace Mpituus4 = pituus4 if cla6e4 == 1 | cla6e4 == 2 | cla6e4 == 3


generate Mpituus5 = .

replace Mpituus5 = pituus5 if cla6e5 == 1 | cla6e5 == 2 | cla6e5 == 3


generate Mpituus6 = .

replace Mpituus6 = pituus6 if cla6e6 == 1 | cla6e6 == 2 | cla6e6 == 3



generate Mpituus7 = .

replace Mpituus7 = pituus7 if cla6e7 == 1 | cla6e7 == 2 | cla6e7 == 3
```

```
generate Mpituus8 = .
replace Mpituus8 = pituus8 if cla6e8 == 1 | cla6e8 == 2 | cla6e8 == 3

generate Mpituus9 = .
replace Mpituus9 = pituus9 if cla6e9== 1 | cla6e9 == 2 | cla6e9 == 3

egen Mpituus = rowtotal(Mpituus*)

*childhood time with biological father = D, codes 1, 4, 5

generate Dpituus0 = .
replace Dpituus0 = pituus0 if ekaperhe == 1 | ekaperhe == 4 | ekaperhe == 5

generate Dpituus1 = .
replace Dpituus1 = pituus1 if cla6e1 == 1 | cla6e1 == 4 | cla6e1 == 5

generate Dpituus2 = .
replace Dpituus2 = pituus2 if cla6e2 == 1 | cla6e2 == 4 | cla6e2 == 5

generate Dpituus3 = .
replace Dpituus3 = pituus3 if cla6e3 == 1 | cla6e3 == 4 | cla6e3 == 5

generate Dpituus4 = .
replace Dpituus4 = pituus4 if cla6e4 == 1 | cla6e4 == 4 | cla6e4 == 5
```

```
generate Dpituus5 = .
replace Dpituus5 = pituus5 if cla6e5 == 1 | cla6e5 == 4 | cla6e5 == 5

generate Dpituus6 = .
replace Dpituus6 = pituus6 if cla6e6 == 1 | cla6e6 == 4 | cla6e6 == 5

generate Dpituus7 = .
replace Dpituus7 = pituus7 if cla6e7 == 1 | cla6e7 == 4 | cla6e7 == 5

generate Dpituus8 = .
replace Dpituus8 = pituus8 if cla6e8 == 1 | cla6e8 == 4 | cla6e8 == 5

generate Dpituus9 = .
replace Dpituus9 = pituus9 if cla6e9== 1 | cla6e9 == 4 | cla6e9 == 5

egen Dpituus = rowtotal(Dpituus*)

browse

*time with stepfather = SD, code 3

generate SDpituus0 = .
replace SDpituus0 = pituus0 if ekaperhe == 3

generate SDpituus1 = .
replace SDpituus1 = pituus1 if cla6e1 == 3
```

```
generate SDpituus2 = .

replace SDpituus2 = pituus2 if cla6e2 == 3


generate SDpituus3 = .

replace SDpituus3 = pituus3 if cla6e3 == 3


generate SDpituus4 = .

replace SDpituus4 = pituus4 if cla6e4 == 3


generate SDpituus5 = .

replace SDpituus5 = pituus5 if cla6e5 == 3


generate SDpituus6 = .

replace SDpituus6 = pituus6 if cla6e6 == 3



generate SDpituus7 = .

replace SDpituus7 = pituus7 if cla6e7 == 3


generate SDpituus8 = .

replace SDpituus8 = pituus8 if cla6e8 == 3


generate SDpituus9 = .

replace SDpituus9 = pituus9 if cla6e9== 3



egen SDpituus = rowlast(SDpituus*)



* time with stepmother = SM, code 5
```

```
generate SMpituus0 = .
replace SMpituus0 = pituus0 if ekaperhe == 5

generate SMpituus1 = .
replace SMpituus1 = pituus1 if cla6e1 == 5

generate SMpituus2 = .
replace SMpituus2 = pituus2 if cla6e2 == 5

generate SMpituus3 = .
replace SMpituus3 = pituus3 if cla6e3 == 5

generate SMpituus4 = .
replace SMpituus4 = pituus4 if cla6e4 == 5

generate SMpituus5 = .
replace SMpituus5 = pituus5 if cla6e5 == 5

generate SMpituus6 = .
replace SMpituus6 = pituus6 if cla6e6 == 5


generate SMpituus7 = .
replace SMpituus7 = pituus7 if cla6e7 == 5

generate SMpituus8 = .
replace SMpituus8 = pituus8 if cla6e8 == 5

generate SMpituus9 = .
replace SMpituus9 = pituus9 if cla6e9 == 5
```

```
egen SMpituus = rowlast(SMpituus*)

*correcting errors

replace Mpituus = 14 if id == 244576000
replace Dpituus = . if id == 244576000
replace SDpituus = . if id == 244576000

replace Dpituus = . if id == 508098000
replace Mpituus = 18 if id == 508098000

browse
keep id Mpituus Dpituus SDpituus SMpituus

save parentpituus_all_N.dta

*merging childhood co-residence data with main data

clear
use anchor2.dta

merge 1:1 id using parentpituus_all_N.dta

drop _merge

*Drop adopted
```

```
drop if hae == 1

drop if amhh1 == 1

drop if avhh1 == 1

*If living with mother (lmhh1 = 1 ) -> igr41p1 = 1 (we live in the same house)

replace igr41p1 = 1 if lmhh1 == 1

*igr41p time to parents dwelling. Missing for biological fathers if they live with mother, give same distance if he2=10 parents live together

replace igr41p3 = igr41p1 if igr41p3 == -3  & he2 == 1

*give stepmother the same distance than stepfathers

replace igr41p4 = igr41p3 if igr41p4 == -3 & hsm1 == 1

*distance to stepfathers

replace igr41p2 = igr41p1 if igr41p2 == -3 & hsv1 == 1

* browse id cla6* cla7* step_asu* stepdadage stepdadtime

*recoding covariates

recode sex_gen (-4=.), gen(sex)

drop if sex == .
```

*ethnicity in two classes

recode ethni (-7=.) (1=0 "German native") (2/5=1 "Other"), gen(ethni2)

*education

recode isced (0=0 "currently enrolled") (1/3=1 "primary and lower secondary") (4/5=2 "upper secondary") (6=3 "post secondary") (7/8=4 "tertiary") (-7=.), gen(educ)

gen highgrade = school if isced == 0

recode highgrade (0=0) (1/3=1) (4/5=2) (6/7=3) (8=.)

recode highgrade (0=0) (1=100) (2=200) (3=300) (-7=.)

egen educ_2 = rowtotal(highgrade educ)

recode educ_2 (0=0 "currently enrolled") (1 100=1 "primary and lower secondary") (2 200=2 "upper secondary") (3 300=3 "post secondary") (4=4 "tertiary") ///

if educ !=., gen(educ_highest)

*age of anchors youngest children

recode ykage (-3 -7 = .), gen(nuorin)

generate nuorin_y = nuorin/12

* number of alive children, 1 if at least one child

recode nkidsbioalv (-1000/-1=.) (1/10=1), gen(kids)

*number of kids cap at 5

recode nkidsbioalv (-1000/-1=.) (1 = 1) (2 = 2) (3 = 3) (4 = 4) (5 6 7 10 = 5), gen(nokids)

*number of biological kids living with anchor

recode nkidsbioliv (-1000/-1=.) (1 = 1) (2 = 2) (3 = 3) (4 = 4) (5 6 7 10 = 5), gen(nokidsliv)

recode nkidsbioliv (-1000/-1=.) (1/10 = 1), gen(kids_home)

* reshaping long format, parent: 1=mother, 2=stepfather, 3=father, 4=stepmother

reshape long igr41p igr68p igr70p, i(id) j(parent)

*igre68 getting help taking care of your children

*igr70 gifts or financial support

*igr41p How much time do you need to get to your X dwelling? (same house=1, less than 10min = 2, ...3h or more=6)

recode igr41p (-4 -3 -1 -2 =.) (1=0) (2=1) (3=2) (4=3) (5=4) (6=5), gen(distance)

*grandparenting questions

recode igr68p (-4 -3 -1 -2 =.) (1=0) (2=1) (3=2) (4=3) (5=4), gen(kidhelp)

recode igr70p (-4 -3 -1 -2 =.) (1=0) (2=1) (3=2) (4=3) (5=4), gen(kidfina)

recode igr68p (-4 -3 -1 -2 =.) (1=0) (2=1) (3=1) (4=1) (5=1), gen(kidhelp2)

recode igr70p (-4 -3 -1 -2 =.) (1=0) (2=1) (3=1) (4=1) (5=1), gen(kidfina2)

```
*PARENT (GP) ika age, page, mage, fage, smage, sfage

*parents' age

generate ika = .
replace ika = mage if parent == 1
replace ika = fage if parent == 3
replace ika = sfage if parent == 2
replace ika = smage if parent == 4
replace ika = . if ika < 0

generate GP = "Z"
replace GP = "Mother" if parent == 1
replace GP = "Stepfather" if parent == 2
replace GP = "Father" if parent == 3
replace GP = "Stepmother" if parent == 4

*generate maternal and paternal lines
generate fline = "X"
replace fline = "MGM" if sex == 2 & parent == 1
replace fline = "MGF" if sex == 2 & parent == 3
replace fline = "PGM" if sex == 1 & parent == 1
replace fline = "PGF" if sex == 1 & parent == 3

replace fline = "SMGM" if sex == 2 & parent == 4
replace fline = "SMGF" if sex == 2 & parent == 2
replace fline = "SPGM" if sex == 1 & parent == 4
replace fline = "SPGF" if sex == 1 & parent == 2
```

```
encode fline, generate(fline2)

describe fline2

* error correction

replace SDpituus = 0 if id == 3255000

replace Dpituus = 17 if id == 3255000

*112050000 typo correction

replace ika = . if id == 112050000 & parent == 3

*412802000  error father age

replace ika = . if id == 412802000 & parent == 3

*cohabitation status

*tabulate relstat

*cohab = 1

recode relstat (-7 =.) (1 2 5 6 7 9 10=0) (3 4 8 11 =1) , gen(cohab)

*Generate chidhood co-residency duration per each parent (M = mother, D = Father, SD = stepfather, stepmother = SM)

generate pituus = .

replace pituus = Mpituus if parent == 1

replace pituus = Dpituus if parent == 3

replace pituus = SDpituus if parent == 2

replace pituus = SMpituus if parent == 4
```

```
* if SD or SM, but no childhood co-residency --> 0

replace pituus = 0 if pituus ==. & parent == 2

replace pituus = 0 if pituus ==. & parent == 4

*Some cases checked individually

replace pituus = 17 if id == 189519000 & parent == 1 // moved with mother?
replace pituus = 17 if id == 53926000 & parent == 1 // moved with mother?
replace pituus = 17 if id == 140628000 & parent == 1 // moved with mother?
replace pituus = 18 if id == 284363000  & parent == 1 // moved with mother and father?
replace pituus = 18 if id == 284363000  & parent == 3 // moved with mother and father?

* he3= 1 parents married, hm1 = mother living status (1 living 2 dead) igr29= mother marital status , igr30 mother have a partner now, igr31 mother lives with partner
*he2 bioparents live together

generate pcohab2 = .

replace pcohab2 = 0 if parent == 1 & igr29 == 1
replace pcohab2 = 0 if parent == 1 & igr29 == 4
replace pcohab2 = 0 if parent == 1 & igr29 == 5
replace pcohab2 = 1 if parent == 1 & igr29 == 3
replace pcohab2 = 1 if parent == 1 & igr29 == 2
replace pcohab2 = 0 if parent == 1 & igr30 == 3
replace pcohab2 = 0 if parent == 1 & igr31 == 2

replace pcohab2 = 0 if parent == 1 & he2 == 2 & hsv1 == 2
```

replace pcohab2 = 1 if parent == 1 & he2 == 1

replace pcohab2 = 1 if parent == 1 & igr30 == 2 & igr31 == 1

*father igr34 marital status igr35 partner now, igr36 lives with partner

replace pcohab2 = 0 if parent == 3 & igr34 == 1

replace pcohab2 = 0 if parent == 3 & igr34 == 4

replace pcohab2 = 0 if parent == 3 & igr34 == 5

replace pcohab2 = 0 if parent == 3 & igr35 == 3

replace pcohab2 = 0 if parent == 3 & he2 == 2

replace pcohab2 = 0 if parent == 3 & igr36 == 2

replace pcohab2 = 0 if parent == 3 & he2 == 2 & hsm1 == 2

replace pcohab2 = 1 if parent == 3 & he2 == 1

replace pcohab2 = 1 if parent == 3 & igr34 == 2

replace pcohab2 = 1 if parent == 3 & igr34 == 3

replace pcohab2 = 1 if parent == 3 & igr35 == 2 & igr36 == 1

replace pcohab2 = 1 if parent == 3 & igr35 == 1

*stepfather

replace pcohab2 = 1 if parent == 2 & igr31 == 1

replace pcohab2 = 1 if parent == 2 & igr29 == 2

replace pcohab2 = 1 if parent == 2 & igr29 == 3

replace pcohab2 = 0 if parent == 2 & igr31 == 2

replace pcohab2 = 0 if parent == 2 & hm1 == 2

*stepmother

replace pcohab2 = 1 if parent == 4 & igr36 == 1

replace pcohab2 = 1 if parent == 4 & igr34 == 2

```
replace pcohab2 = 1 if parent == 4 & igr34 == 3
replace pcohab2 = 0 if parent == 4 & igr36 == 2

replace pcohab2 = 0 if parent == 4 & hv1== 2


*parents not together

replace he1 = 2 if he1 == .

*remove unnecessary parent lines

drop if parent == 1 & hm1 == 2

drop if parent == 1 & hm1 == .
drop if parent == 2 & hsv1 == 2
drop if parent == 2 & hsv1 == .
drop if parent == 3 & hv1 == 2
drop if parent == 3 & hv1 == .
drop if parent == 4 & hsm1 == 2
drop if parent == 4 & hsm1 == .

* drop youngest cohort, too few obs with children

drop if cohort == 1

*browse id pcohab2 parent hm1 hv1 hsv1 hsm1 hm2 hv2 hsv2 hsm2 igr29 igr31 igr36 igr34  he2 sort parent


* hm2 hv2 hsv2 hsm2 contact with parent
```

```
*include only those who have at least one child

drop if kids == 0
drop if kids == .


*combination of some variables because too few obs otherwise

recode educ_highest (0 = 1)

recode nokids (5 = 4)

recode nokids (4 5 = 3)

recode kidfina (4 = 3), gen(kidfina4)
recode kidhelp (4 = 3), gen(kidhelp4)

recode kidfina (2 = 1) (3 = 2) (4 = 2), gen(kidfina3)
recode kidhelp (2 = 1) (3 = 2) (4 = 2), gen(kidhelp3)


label define kidfina3_l 0 "Never" 1 "Sometimes" 2 "Often"
label values kidfina3 kidfina3_l

label define kidhelp3_l 0 "Never" 1 "Sometimes" 2 "Often"
label values kidhelp3 kidhelp3_l

*missing obs
misstable summarize pcohab2 distance pituus educ_highest nokids cohab ethni2 nuorin_y, generate(_miss)

ssc install mdesc
```

```
mdesc pcohab2 distance pituus educ_highest nokids cohab ethni2 nuorin_y

*delete missing information

drop if kidhelp == .
drop if kidfina == .

drop if pcohab2 == .
drop if distance == .
drop if pituus == .
drop if educ_highest == .
drop if nokids == .
drop if cohab == .
drop if ethni2 == .
drop if nuorin_y == .

*partial proportionality for ordered logistic regression did not hold ---> Partial Proportional Odds Model

*Financial support given to (step)grandchildren

gologit2 kidfina3 ib2.fline2 i.cohort i.ethni2 i.educ_highest i.distance i.cohab i.pcohab2   i.nokids c.nuorin_y
, robust cluster(id) autofit gamma
estat ic
test
margins i.fline2
marginsplot

*model without covariates

gologit2 kidfina3 ib2.fline2, robust cluster(id) autofit gamma
```

*model with weight

gologit2 kidfina3 ib2.fline2 i.cohort i.ethni2 i.educ_highest i.distance i.cohab i.pcohab2 i.nokids c.nuorin_y [weight=dweight], robust cluster(id) autofit gamma

*Help in childcare

gologit2 kidhelp3 ib2.fline2 i.cohort i.ethni2 i.educ_highest i.distance i.cohab i.pcohab2 i.nokids c.nuorin_y, robust cluster(id) autofit gamma

estat ic

margins i.fline2

marginsplot

*model without covariates

gologit2 kidhelp3 ib2.fline2, robust cluster(id) autofit or gamma

*model with weigh

gologit2 kidhelp3 ib2.fline2 i.cohort i.ethni2 i.educ_highest i.distance i.cohab i.pcohab2 i.nokids c.nuorin_y [weight=dweight], robust cluster(id) autofit gamma

*Variable indicating step-grandparent

generate stepparent = .

replace stepparent = 1 if parent == 2

replace stepparent = 2 if parent == 4

*Financial support given to (step)grandchildren and co-residence duration (pituus)

gologit2 kidfina3 i.stepparent i.sex c.pituus i.cohort i.ethni2 i.educ_highest i.distance i.cohab i.pcohab2 i.nokids c.nuorin_y, autofit gamma

```
margins, at(pituus=( 0 1 2 3 4 5 6 7 8 9 10 11 12 13 14 15 16 17) )

marginsplot

*without covariates

gologit2 kidfina3 i.stepparent c.pituus, autofit or gamma

margins, at(pituus=( 0 1 2 3 4 5 6 7 8 9 10 11 12 13 14 15 16 17) )

marginsplot

*model with weight

gologit2 kidfina3 i.stepparent i.sex c.pituus i.cohort i.ethni2 i.educ_highest i.distance i.cohab  i.pcohab2
i.nokids  c.nuorin_y [weight=dweight], autofit  gamma

margins, at(pituus=( 0 1 2 3 4 5 6 7 8 9 10 11 12 13 14 15 16 17) )

marginsplot

*Help in childcare and and co-residence duration (pituus)

gologit2 kidhelp3 i.stepparent i.sex c.pituus i.cohort i.ethni2 i.educ_highest i.distance i.cohab  i.pcohab2
i.nokids  c.nuorin_y, autofit gamma

margins, at(pituus=( 0 1 2 3 4 5 6 7 8 9 10 11 12 13 14 15 16 17) )

marginsplot

*model with weight

gologit2 kidhelp3 i.stepparent i.sex c.pituus i.cohort i.ethni2 i.educ_highest i.distance i.cohab  i.pcohab2
i.nokids  c.nuorin_y [weight=dweight], autofit gamma

 margins, at(pituus=( 0 1 2 3 4 5 6 7 8 9 10 11 12 13 14 15 16 17) )

marginsplot
```

```
*Variable for separated grandparents

generate eroparent = .
replace eroparent = 1 if parent == 1 & he2 == 2
replace eroparent = 2 if parent == 3 & he2 == 2

*variable for weight
*[weight=dweight]

*Financial support given to (step)grandchildren and co-residence duration (pituus)

gologit2 kidfina3 i.eroparent i.sex c.pituus i.cohort i.ethni2 i.educ_highest i.distance i.cohab  i.pcohab2 i.nokids  c.nuorin_y,  autofit gamma
margins, at(pituus=( 0 1 2 3 4 5 6 7 8 9 10 11 12 13 14 15 16 17 18) )
marginsplot

*Help in childcare and  co-residence duration (pituus)

gologit2 kidhelp3 i.eroparent i.sex c.pituus i.cohort i.ethni2 i.educ_highest i.distance i.cohab  i.pcohab2 i.nokids  c.nuorin_y , autofit gamma
margins, at(pituus=( 0 1 2 3 4 5 6 7 8 9 10 11 12 13 14 15 16 17 18) )
marginsplot

margins sex
```
